# Eighteenth-century science communication? Exploring the case of Julien Offray de La Mettrie

**DOI:** 10.1177/09636625261441408

**Published:** 2026-05-04

**Authors:** Felix Eichbaum

**Affiliations:** 1 Karlsruhe Institute of Technology (KIT), Germany

Julien Offray de La Mettrie (1709–1751), a French physician and philosopher, stands as one of the most provocative and systematically marginalized figures of the Enlightenment. Alternately discredited as the radical materialist *Monsieur Machine* and praised as a visionary medical reformer, La Mettrie’s career and reputation have been constantly shaped by the ideological lenses through which he has been read (Cryle, 2006; Jauch, 2012; Wellman, 1992). One aspect of his legacy, however, has received little to no attention yet: his role as popularizer and communicator of science. To explore this gap, three strands of La Mettrie’s activity are examined: his translation of scientific texts, his satirical engagement with societal and scientific discourse, and his contributions to public health advocacy. Building on the premise that historical strategies of science popularization can inform contemporary science communication, it is the aim of this study to relate La Mettrie’s approaches to modern concepts, thereby bridging historical analysis and current practice in the field.

## 1. Life and times of La Mettrie: The emergence of science popularization

La Mettrie lived during a decisive period of scientific inquiry and communication in Europe, spanning the early to mid-eighteenth century. Born in France, he studied in Paris and Rennes, trained under the renowned physician Herman Boerhaave in the Dutch city of Leiden, and resided his final years at the court of Frederick II in Potsdam, Germany (Laska, 2008: 65–66). His lifetime coincided with a transformative phase in European history, marked by the emergence of a bourgeois public sphere, an increasing societal interest in scientific knowledge, and the advent of early public science popularization.

Throughout the eighteenth century, novel spaces for public discourse, exchange of ideas, and educated conversation emerged across Europe in the form of coffee houses, salons, or reading societies, manifesting the decline of an exclusive courtly aristocratic public sphere (Habermas, 1991). The emerging public sphere of the salons consisted of all individuals who could, in principle, participate as readers, listeners, or spectators in ongoing debates while those actually participating served as the “bourgeois representation” of this potential public (Habermas, 1991: 51). Concurrently, an increasing number of science-adjacent books, scholarly journals and newspapers began to appear. A foundational work of this early popular scientific writing is the 1686 publication *Entretiens sur la pluralité des mondes* by La Mettrie’s contemporary Bernard Fontanelle, in which Copernican astronomy is presented in an accessible dialogue format written in French rather than Latin (Daum, 2002: 265). This “publicization of knowledge” was crucial not only in constituting a scientifically interested public but also in weakening barriers between the scientific elite and other social classes (Cooter and Pumfrey, 1994). As a young adult, La Mettrie also witnessed the rise of the first professional science popularizers in France, whose activities included the public demonstration and replication of major scientific experiments, thereby laying the groundwork for what became a recognized profession by the time of the French Revolution (Lynn, 2006: 19–25). This increase in the societal relevance of scientific knowledge corresponded with the expansion of scientific institutions, such as state-funded academies and *musées*, which were less exclusive than academies yet more formal than reading societies, coffee houses, or salons, and similarly dedicated to serving discussion and knowledge dissemination (Lynn, 1999).

These social and institutional changes, however, did not occur without friction. La Mettrie operated in a socio-political environment marked by rigid social stratification and religious persecution following the Revocation of the Edict of Nantes (1685), while a growing awareness of these inequities created a social climate where traditional authorities were increasingly viewed with suspicion (Adams, 1991; Darnton, 1982; Roche, 1998). It is within this landscape of growing public interest in science and social fractures rooted in religious exclusion and rising class consciousness that La Mettrie’s communicative approaches become intelligible. Much like his famous contemporaries Voltaire (1694–1778) and Émilie du Châtelet (1706–1749), he sought to engage a broader, more diverse readership in scientific and philosophical debates; yet, he distinguished himself through a more confrontational and provocative attitude, earning him the reputation of a radical outlier rather than a serious contributor to public enlightenment. Challenging that label, this study seeks to reposition La Mettrie as a serious but unconventional pioneer of early science communication.

Considering the aforementioned developments from a contemporary standpoint, however, it would be anachronistic to categorize such early practices and concerns as examples of professional science communication. While they already foreshadow modern practices, the crucial developments in the realms of science, media, and society that enabled the emergence and professionalization of the field occurred primarily in the nineteenth and twentieth centuries (Hanauska, 2020; Hochadel, 2020). Accordingly, La Mettrie’s approaches to science communication should be understood as activities that prefigure rather than embody that category. Moreover, recognizing that the term “science popularization” has been criticized for implying a hierarchical view on knowledge transfer between learned scientists and an ignorant public (Cooter and Pumfrey, 1994; Hanauska, 2020; Hilgartner, 1990), this study employs a broader, more inclusive understanding of the term – one that acknowledges the dynamic interactions between knowledge production and reception, framing audiences not only as passive recipients but also as active participants in the co-construction of scientific meaning. La Mettrie’s communicative approaches appear to resonate with such principles, as he frequently sought to relate abstract scientific concepts to the lived experiences and practical reasoning of his audiences.

## 2. Mediating knowledge: La Mettrie’s threefold approach towards science popularization

### La Mettrie as translator and commentator

Following his medical studies and doctorate, La Mettrie travelled to Leiden in 1734 to study under the renowned clinician Hermann Boerhaave. There, he not only deepened his clinical training but also translated several of Boerhaave’s foundational works from Latin into French, increasing their accessibility from a small circle of professionals to broader parts of society. Yet, he did not limit himself to mere translation: his edition of Boerhaave’s *Institutiones medicae* included a substantial commentary spanning six additional volumes (Wellman, 1992: 107). Such editorial interventions were not uncommon; prefaces, commentaries, footnotes, and other paratextual elements were regularly employed by translators to showcase their scholarship or to capitalize on an original author’s prominence to establish themselves as independent thinkers (Gipper and Stefanelli, 2021: 179).

What distinguishes La Mettrie’s commentary on Boerhaave is its clear orientation towards a different target audience than the one originally envisioned, as his version of the *Institutiones* is directed at a popular audience, written in a casual manner with plenty anecdotes from the Parisian scene of that time (Wellman, 1992: 111). These differences in style and target audience reflect the broader structural transformation of the scientific landscape and the public sphere in eighteenth-century Europe: a shift from the exclusive Latin-speaking academic elite to a vernacular, socially diverse public sphere of the scientifically interested. Whereas Boerhaave, born in 1668, still addressed a strictly professional readership, La Mettrie responded to these changing social conditions by engaging new audiences, borrowing Boerhaave’s authority to promote his own medical and philosophical perspectives to the public.

Consequently, La Mettrie’s translations and commentaries of scientific texts contributed to the popularization of medical and scientific knowledge in a way that resembles modern practices of translating across language and levels of expertise, both focusing on the strategic use of language as a means for facilitating effective and targeted science communication (Krieger and Gallois, 2017; Márquez and Porras, 2020). His approach foreshadows current understandings of translation not merely as linguistic transfer, but as a sociocultural process that inevitably affects the original scientific information (Ødemark and Engebretsen, 2022). By contextualizing complex information within the lived experience of the reader and through strategically adapting language, style, and content, La Mettrie leveraged fundamental mechanisms for making scientific knowledge both accessible and actionable across diverse audiences.

### La Mettrie as satirist and pamphleteer

During La Mettrie’s student years from 1727 to 1731, Paris witnessed a veritable pamphlet war between physicians and surgeons, in which he became an active participant shortly after completing his formal education (Wellman, 1992: 34). At the core of this controversy were questions of professional and social inequality between physicians and surgeons, as well as divergent training methods within the medical faculty. Generally concerned with public health, La Mettrie sided with the surgeons, whose empirical, practice-based training he deemed more useful and oriented towards the common good than the theoretical instruction offered at the university. In support of their cause, he authored a total of seven volumes of satirical commentary on contemporary medicine. His provocative style, frequently employing biting irony and mockery, combined with the socially relevant nature of the debate, enabled La Mettrie to reach a wider public and effectively draw attention to the shortcomings of medical education and practice of the time (Wellman, 1992: 35–43). It is important to recognize, however, that communicative provocation is a double-edged sword. While La Mettrie’s wit successfully gained him public interest, it also alienated parts of his readership, including members of the very institutions he sought to reform, ultimately leading to the loss of his patronage and institutional support (Christensen, 1996: 121).

While satire and pamphlets generally vary widely in length, content, and form, they are united by their engagement with controversial social issues and their explicit intent to influence public opinion (Chisick, 1993: 149). Disseminated through town meetings, sermons, and everyday conversations, pamphlets reached significantly wider and more socially diverse audiences than most other printed materials of the time (Sawyer, 1990: 69). Utilizing this format, La Mettrie succeeded in lifting medical discourses out of the professional sphere and into the public domain, using wit and irony to make complex, socially relevant topics accessible to a non-specialist readership. Among these are his *Politique du médecin de Machiavel, ou le chemin de la fortune ouvert aux Médecins* (1746), earning him the label of a “full-blown polemicist” (Wellman, 1992: 44), and *La Faculté vengée* (1747) published as a satirical comedy in three acts to explicitly criticize the Parisian Faculty of Medicine.

From a contemporary perspective, La Mettrie’s approach to facilitating public discourse through creative and provocative formats resembles specific practices of modern science communication, such as Science Slams (Niemann et al., 2020) or arts-based methods like theatre performances (Weitkamp and Almeida, 2022). These formats foster public engagement, reach broader audiences, and trigger individual reflection (Achiam et al., 2026; Fraaije et al., 2022). By using “edutainment,” humour and intellectual provocation as epistemic tools, they – much like La Mettrie’s own writings – seek not only to convey knowledge but also to challenge audiences’ assumptions and stimulate critical reflection. Rather than presenting medical knowledge as self-evident and unquestionable, La Mettrie emphasizes its empirical and tentative character, encouraging his readers to “[b]reak the chain of your prejudices, arm yourselves with the torch of experience” (La Mettrie JOd, 1912: 146), thereby actively fostering their capacity for independent thought.

### La Mettrie as advocate of social medicine

At first glance, it may seem surprising that the traditionally trained physician La Mettrie sided with the surgeons during the Parisian pamphlet war, given that the Paris Faculty of Medicine regarded them, at best, as modestly educated practitioners, if not unscrupulous charlatans (Wellman, 1992: 35). To understand this position, one must consider the historical distinctions between the professional roles and social status of physicians and surgeons during La Mettrie’s time. In Ancien Régime France, where access to professional status was heavily gatekept by religious and class conformity, physicians were typically part of the high bourgeoisie, caring primarily for patients from similar elite circles who often served as their patrons. In contrast, surgeons tended to work with – and belonged to – a broader cross-section of the population, including the lower classes. These differences were also reflected in their respective training: while physicians were educated through theoretical, text-based instruction at universities, surgeons acquired knowledge through direct clinical practice, often in the context of military campaigns (Wellman, 1992: 16–21).

In a sense, La Mettrie acted as a “go-between” (Raj, 2016; Schaffer et al., 2009) in this regard, bridging the distinct roles of physicians and surgeons, not only through his writings but also during his career. Conventionally trained as a doctor he was appointed head physician of the Duke of Gramont’s regiment in 1742. In this position, he had to prove his medical competence on the battlefield during the War of the Austrian Succession, working side by side with surgeons. Marked by these wartime experiences, La Mettrie increasingly valued the practical expertise of surgeons over what he perceived as the self-righteous ignorance of his affluent Parisian colleagues. His growing critique of these doctors is reflected in the early outlines of his philosophical debut, *Histoire naturelle de l’âme*, whose materialist tendencies can be traced back to his working in military hospitals (Hacking, 2009). Subsequently, La Mettrie directed particular critical attention to the Paris Faculty of Medicine, which he identified as a structural root of the deteriorating metropolitan medical care due to its efforts to restrict the number of physicians to preserve their social status and privileges (Wellman, 1992: 52).

This criticism of the health system was already present in La Mettrie’s early medical writings, which Jauch (1998) describes as a testament to his social-medical vision and his high medical ethos (p. 78). These writings included not only enlightening – and at times scandalous – passages on sexual hygiene and epidemic prevention but also general advice akin to what would today be termed modern health education. In addition, he published a series of short articles in popular journals, the *Lettres sur l’art de conserver la santé*, written with the explicit aim “to inform his fellow citizens so that they would be able to know what ought to be required of the physician and to have some control over their own state of health” (Wellman, 1992: 96).

Looking at the broader picture, La Mettrie’s efforts towards public health education and systemic reform contribute to both the reformist framework of the Enlightenment and the dissemination of scientifically grounded information of social relevance. From today’s perspective, his advocacy of social medicine and his efforts to disseminate medical information to broader segments of society resembles current initiatives to reach underserved audiences with science communication (Dawson, 2018; Humm et al., 2020). Although his motivation stemmed more from an Enlightenment critique of institutional authority rather than from modern discourses on inclusion, his work nonetheless prefigures the emancipatory potential that contemporary science communication seeks to fulfil. It is this attitude towards scientific knowledge as a public good, and the awareness of science communication’s potential for social empowerment, that reflects La Mettrie’s commitment to what we now recognize as the democratization of expert knowledge and the foundations of social medicine.

## 3. Conclusion

La Mettrie’s practices of popularization – particularly his strategic use of language, his employment of creative and provocative formats, and his framing of scientific knowledge as a public good – remain relevant to current scholarship in science communication. Although deeply embedded in the socio-political context of eighteenth-century Europe, they still resonate with contemporary efforts to make scientific discourse more accessible, engaging, and socially responsible. Looking back at La Mettrie’s case illustrates how inclusivity in science communication can function not as an optional add-on, but as an inherent aspect that is reflected in the audiences addressed, the formats used, and the language chosen. Moreover, his early commitment to medical reform demonstrates both the value of engaging with pressing societal issues and the courage required to step out of the relative comfort of scholarly debate into the complexities of public discourse.

Methodologically, this study highlights the value of viewing historical case studies through a modern lens, thereby shedding light on a previously understudied facet of La Mettrie’s work and tracing conceptual continuities from early forms of popularization to contemporary ideals of inclusive engagement. While social structures, scientific institutions, and media landscapes have evolved fundamentally since La Mettrie’s time, the enduring relevance of his threefold approach towards public engagement underscores his pioneering role in what might be anachronistically described as eighteenth-century science communication. As La Mettrie JOd (1912) himself might say: “Dispute it now who will” (p. 149).
